# Possible Mechanisms of Biological Effects Observed in Living Systems during ^2^H/^1^H Isotope Fractionation and Deuterium Interactions with Other Biogenic Isotopes

**DOI:** 10.3390/molecules24224101

**Published:** 2019-11-13

**Authors:** Alexander Basov, Liliya Fedulova, Ekaterina Vasilevskaya, Stepan Dzhimak

**Affiliations:** 1 Department of Fundamental and Clinical Biochemistry, Kuban State Medical University, Krasnodar 350063, Russia; son_sunytch79@mail.ru; 2 Department of Radiophysics and Nanotechnology, Kuban State University, Krasnodar 350040, Russia; 3 The V.M. Gorbatov Federal Research Center for Food Systems of Russian Academy of Sciences, Moscow 109316, Russia; ranatemporalia@mail.ru (L.F.); e.vasilevskaya@fncps.ru (E.V.); 4 Federal Research Center the Southern Scientific Center of the Russian Academy of Sciences, Rostov-on-Don 344006, Russia

**Keywords:** deuterium, nonradioactive isotopes, neutron, isotopic resonance, neurodegenerative diseases, electron density delocalization, isotopic discrimination, mitochondrial disorders, living systems

## Abstract

This article presents the original descriptions of some recent physics mechanisms (based on the thermodynamic, kinetic, and quantum tunnel effects) providing stable ^2^H/^1^H isotope fractionation, leading to the accumulation of particular isotopic forms in intra- or intercellular space, including the molecular effects of deuterium interaction with ^18^O/^17^O/^16^O, ^15^N/^14^N, ^13^C/^12^C, and other stable biogenic isotopes. These effects were observed mainly at the organelle (mitochondria) and cell levels. A new hypothesis for heavy nonradioactive isotope fractionation in living systems via neutron effect realization is discussed. The comparative analysis of some experimental studies results revealed the following observation: “Isotopic shock” is highly probable and is observed mostly when chemical bonds form between atoms with a summary odd number of neutrons (i.e., bonds with a non-compensated neutron, which correspond to the following equation: Nn − Np = 2k + 1, where k ϵ Z, k is the integer, Z is the set of non-negative integers, Nn is number of neutrons, and Np is number of protons of each individual atom, or in pair of isotopes with a chemical bond). Data on the efficacy and metabolic pathways of the therapy also considered ^2^H-modified drinking and diet for some diseases, such as Alzheimer’s disease, Friedreich’s ataxia, mitochondrial disorders, diabetes, cerebral hypoxia, Parkinson’s disease, and brain cancer.

## 1. Introduction

It is well known that many physical and chemical processes in living systems are accompanied by isotope fractionation among the atoms of biologically significant elements, primarily H, C, O, and N [1,2]. Changes in the ratio of heavy and light isotopes of biogenic elements cause quantum (tunnel), thermodynamic, and consequently kinetic isotope effects that manifest themselves under natural conditions in acceleration, deceleration, or branching metabolic reactions, as well as in the changed speed of metabolite influx through transport channels; this leads to a local fluctuation in the pool of biologically active substances [3] in some compartments or organelles of the cells. Moreover, the change in the natural isotope ratio in some structural components of living systems is accompanied by the modification of some biochemical reaction mechanisms in animals and plants, which is caused, for example, by compartmentalization [4]. in general, this can lead, among other things, to faster adaptation under the influence of various stress factors.

Some manifestations of the isotope effects of certain elements under natural conditions have been studied and published [5]. However, it is extremely important to know the mechanisms of isotope effects to understand the specific features and reconstruction mechanisms of physiological processes in plants and animals, depending on their geographical origin and habitat [6]. This approach makes it possible to use different isotope concentration values obtained from studies to characterize biological objects [7], including clarification of the specific features of biochemical processes and reaction mechanisms, as well as the reconstruction of climatic, physiological, ecological, and environmental conditions during a certain period in the vital activities of organisms [8,9,10].

However, despite considerable amounts of experimental data on changes in the ratio of light and heavy isotopes of biogenic elements in biological objects depending on the habitat [11,12], possible mechanisms of the influence of nonradioactive isotopes of hydrogen, oxygen, carbon, and others on living objects remain unexplained in the study of their exchange, especially for combined (poly-isotopic) effects. Their explanation is necessary to clarify isotope action regularities and predict ultimate biological effects in organisms under the influence of naturally or artificially generated fluctuations of isotopic composition, and to create new methods for (poly-)isotopic correction of metabolic abnormalities observed in the body when exposed to various stress factors (chemical, physical [13], or emotional [14]).

This issue is of current interest, as there are now opportunities to correct the ratio of light and heavy isotopes of biogenic elements in the body via modification of the isotope composition of food and water [15,16,17]. It must be noted that changes in the isotope composition of food products can occur in both directions—increases and decreases in their heavy isotope concentrations [18,19]. The latter is especially relevant owing to the possible influence of differences in the ratio of light and heavy isotopes of hydrogen on the adaptive potential of an organism [20,21,22,23], which seems promising with regard to corrective measures that are taken in biology and medicine to prevent disadaptation of biological objects under unfavorable existence conditions. Prediction of changes in the magnitude and direction of the isotope gradient under particular physiological and pathological conditions is especially important because of different isotope exchange rates in the tissues of internal organs with different metabolic activities, owing to gender, age, and temperature differences in individuals of the same species [24]. Therefore, purposefully changing the heavy and light isotope ratio (e.g., deuterium/protium) in biological media and internal organs can provide an opportunity for a preventive increase in the adaptive potential of an organism in case of a developing pathological process. This is possible by modifying the intensity of the metabolic processes and structural rearrangements at the cellular level in living beings, which are both explained by potentially the most profound kinetic isotope effect, arising as hydrogen atoms are fractioned owing to differences in their mass (^1^H и ^2^H), and an expected positive biological impact on the body caused by the removal of heavy isotopes with pronounced negative kinetic isotope effects [25].

Another major discovery is proof of the presence of paramagnetic isotope effects in some metals (calcium, magnesium, and zinc) taking part in biocatalysis [26,27,28,29,30,31], which show a change in the activity of enzymes regulating energy exchange and the transfer of genetic information in cells, depending on the isotope composition of the medium [32,33].

Therefore, the purpose of this study was to substantiate possible formation mechanisms of paradoxical biochemical and biophysical effects arising from the interaction of deuterium or protium with other isotopes and combinations of various macro- and microelements in living systems.

For a more complete understanding of the effects observed when individual fractions of heavy or light isotopes accumulate, the key mechanisms for the realization of their bioeffects should be considered. This approach requires detailed analysis, as it has been shown by different scientific groups that metabolic processes in the body can be influenced (stimulated or inhibited) by both decreases and increases within the same range in the amount of heavy isotopes in comparison with their natural levels in the environment [34,35,36,37,38].

## 2. Molecular Effects of ^2^H/^1^H Exchange

Thermodynamic disparity of isotopic compounds can lead to uneven distributions of hydrogen isotopes when equilibrium is reached because of isotope exchange (^2^H/^1^H) reactions among cells, as well as the predominant accumulation of one of the isotopic forms in intra- or intercellular space. Isotope exchange reactions in biological systems can be accompanied by a change not only in thermodynamic characteristics (diffusion coefficient and specific charge of ions), but also primarily in kinetic ones (rate of biochemical reactions) at the molecular level. It is known that the replacement of deuterium by protium in high molecular compounds does not always affect their thermodynamic state significantly. Nevertheless, some works describe an intermolecular isotope effect caused by a more stable interaction between the deuterated solvation shell and the biomolecule itself (protein, DNA, etc.); this is accompanied by a decrease in their involvement in biochemical reactions due to the delayed desolvation of some regulatory parts of molecules during their transition into a functionally active state [39]. However, during selective changes in active and allosteric centers of enzymes associated with hydrogen isotope exchange as part of readily dissociating groups (hydroxyl (–OH), thiol (–SH), primary protonated, and secondary amino groups (–NH_3_^+^, =NH)), increasing the amount of protium can change the rate of catalytic processes through a decrease in the energy of the molecule’s transition state activation during biocatalysis reactions. It should be noted that in living organisms, intermolecular and intramolecular kinetic isotope effects will be combined, owing to a complex organization of high-molecular compounds (proteins and nucleic acids) that have high solvation ability, and consequently because of hydrogen isotope exchange between dissociating groups in the macromolecule and its hydration shell. Such changes not only lead to the modification of energy interactions between enzymes and substrates (intermolecular effects) but can also be accompanied by intramolecular conformational rearrangements at different rates; this occurs in “enzyme–substrate” or “enzyme–cofactor (coenzyme)” complexes, which can shorten some stages of biocatalytic transformations or accelerate the transition of the enzyme into an active form after the completion of a catalytic cycle, thereby increasing the activity of biochemical processes.

It should be noted that the primary kinetic isotopic effect is characterized by an increase in the energy (En) of rupture for bonds formed by heavier isotopes, for example, En_rupture(A–D)_ > En_rupture(A–H)_ (where A is atom). In some studies, attempts were made to extrapolate the data obtained in vitro to in vivo systems [40,41]. 

**Figure 1:**
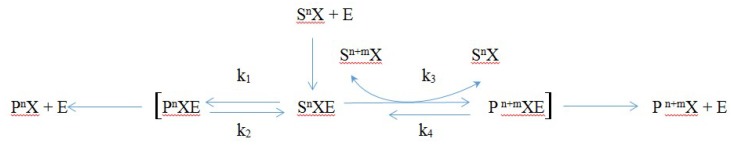
Reaction 1: Fractionation of isotopes (atomic mass ^n+m^X > atomic mass ^n^X) is observed at k_1_ ≈ k_2_; k_3_ >> k_4_; k_3_ >> k_1_. Note: S—(bio-)chemical substrate; P—product of (bio-)chemical reaction; E—enzyme (catalyst).

**Figure 2:**
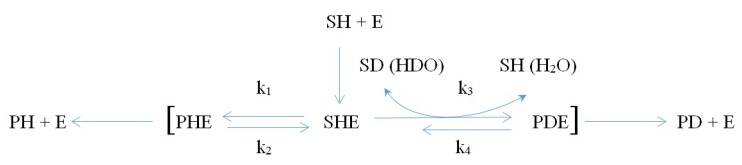
Reaction 2: Fractionation of deuterium is observed when k_1_ ≈ k_2_; k_3_ >> k_4_; k_3_ >> k_1_. Note: S—(bio-)chemical substrate; P—product of (bio-)chemical reaction; E—enzyme (catalyst).

**Figure 3:**
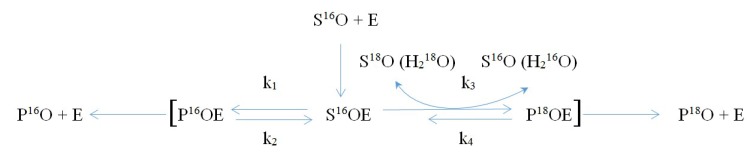
Reaction 3: Fractionation of oxygen isotope ^18^O is observed when k_1_ ≈ k_2_; k_3_ >> k_4_; k_3_ >> k_1_. Note: S—(bio-)chemical substrate; P—product of (bio-)chemical reaction; E—enzyme (catalyst).

Some particularities of various isotope fractionations, including oxygen and hydrogen, observed during biochemical reactions are shown below; these account for the possible uneven local distribution of isotopes, not only within parts of the body, but also within some cells and even organelles with relatively isolated compartments (Reactions 1–3, Figure 1, Figure 2 and Figure 3).

Isotope fractionation in enzyme systems can occur in activated enzyme–substrate complexes (such as S^n^XE in Reaction 1, Figure 1), when the amount of energy required to replace light isotopes with heavy isotopes decreases significantly. Moreover, for a more energetically favorable decomposition of the enzyme–substrate complex with heavy isotopes, a substantial shift of the reaction toward the formation of a product containing a heavy isotope (P^n+m^X) will be observed. This will be pronounced in case of reversibility of the transitional state in the [P^n^XE ↔ S^n^XE] system, when the rate of the forward reaction is the same as that of the reverse reaction; however, this rate should be much lower than that of the direct reaction in the transitional state of the [S^n^XE ↔ P^n+m^XE] system, which will lead to a significant increase in the concentration of heavy isotopes in the reaction products. The described mechanisms were confirmed by the presence of the revealed similar intramolecular rearrangements (rapid keto–enol isomerizations) in the photodissociation of acetaldehyde enriched with deuterium (C^2^H_3_C^1^HO [42]):

^2^H_2_C^2^H–C^1^H=O → [^2^H_2_C=C^1^H–O^2^H ↔ ^2^H_2_C=C^2^H–O^1^H ↔ ^2^HC^1^H=C^2^H–O^2^H] · n → ^2^H_2_C^1^H–C^2^H=O, for *n* ≥ 20 (number of keto–enol isomerizations).

Considering that the energy required for intramolecular rearrangements in the enzyme–substrate complex is lower than the energy required for keto–enol isomerizations, the probability of heavy isotope fractionation significantly increases in proportion to the number of rearrangements in the [P^n^XE ↔ S^n^XE] system, which indicates a high probability of heavy isotope fractionation under natural conditions.

It should be noted that an additional possibility of fractionation is observed for heavy isotopes of hydrogen and oxygen that are part of an activated enzyme–substrate complex due to fixation of ^2^H and ^18^O from the solvent (water: HDO [39,43,44] or H_2_^18^O), in which all biochemical processes (Reactions 2 and 3, Figure 2 and Figure 3) proceed. The latter can lead to a more significant and widespread fractionation of these isotopes in living systems [1,45,46,47,48].

Moreover, it was described that the solubility of individual active pharmaceutical ingredients (such as bendazole hydrochloride, series Р111106) differs about by 50% in natural water (δ^2^H = −101‰) and deuterium-depleted water (δ^2^H = −974‰ [49]). In some studies, the influence of deuterium-depleted water (δ^2^H = −679‰) on the efficiency of protein extractions (where these proteins were from 0.11 to 0.2 parts of the total protein material, including members of the family α- and β-chains of hemoglobin and actin-binding protein (profilin 1)) from the spleen, thymus, and lymph nodes of *Sus scrofa* was also shown [50,51,52,53], and this approach was allowed to receive more active livestock products. 

## 3. Isotope Exchange Effects Observed at the Organelles

The abovementioned influence of the rates of biochemical processes at different ratios of light and heavy isotopes of biogenic elements on the functioning of organelles and subcellular structures is evident in the evaluation of energy metabolism intensity in mitochondria, which makes it possible to characterize the resulting impact of isotope fluctuations on metabolism [54,55,56]. In this case, the replacement of deuterium by protium leads, according to a number of studies, is due to the acceleration of proton fluxes in mitochondria; consequently, this leads to an increase in the production of some substrates, providing, among other things, a higher energy exchange, thereby increasing the resistance of the cell to negative external or internal effects (for example, to hypoxia and intoxication). The effect of isotope exchange on catalytic complexes in some organelles (mitochondria [57], peroxisome [58,59], and lysosome) can change the intensity of metabolic processes at the cellular level as well as substantially modify the resistance or response of biological tissue.

The latter is explained by the fact that one of the key features of metabolism in eukaryotic cells is the compartmentalization of reactions in different organelles. The most critical effect of ^2^H/^1^H isotope exchanges is the mitochondria generating and using universal reducing substrates that are widely involved in redox reactions in both the organelle itself and the cytosol, supporting biosynthesis, redox homeostasis, transduction of intracellular signals, adenosine triphosphate generation, etc. Moreover, deuterium atoms transferred, for example, as part of carbohydrates, because of metabolic transformations associated with NADH_2_ and NADPH + H systems, can migrate from the cytosol and back; they enter amino acids, fatty acids, and the pentose–phosphate backbone of nucleic acids, thereby enriching the pool of heavy isotopes in organic compounds, which is also a special case of ^2^H/^1^H fractionation at the cellular level [60,61].

A cell in a varied functional state is characterized by a specific level of energy and biosynthetic needs that can vary within a certain range, modifying the chains of metabolic transformations with pronounced competition for the pyruvic acid fund; this is accompanied by a change in the proportions of parts of the pyruvic acid fund selectively extracted for the energy system of the cell and metabolite synthesis, causing isotope selection [62]. In addition, metabolic water can be intensively produced in the peroxisomes under particular conditions [59], which also creates auxiliary possibilities for the fractionation of non-radioactive hydrogen isotopes.

## 4. In Vivo ^2^H/^1^H Exchange Effects

Changes in the ^2^H/^1^H isotope composition in living systems can often be caused by different ratios of deuterium and protium in drinking water and food; these changes are observed both during the migration of living organisms and consumption of food produced artificially with reduced amounts of heavy nonradioactive isotopes of biogenic elements [1,2,5], mostly deuterium (for example, deuterium-depleted water).

Fluctuations in the isotope composition at the organism level, and probably, the formation of a new ^2^H/^1^H isotope gradient opposite to the physiological gradient (δ^2^H “visceral organs” > δ^2^H “blood plasma”), can lead to an increase in the activity of humoral and cellular protective systems; this causes a nonspecific phenomenon of increasing resistance of the organism because of preconditioning, during which protective mechanisms at the cellular level are potentiated and information transfer through secondary messengers occurs [2,23].

The described processes can lead to an increase in the rate of transcription and synthesis of heat shock proteins and antioxidant enzymes (for example, superoxide dismutase and catalase), an increase in the content of antinociceptive factors and low-molecular-weight reducing equivalents of the antioxidant system, and changes in the production of reactive oxygen species and free radicals, activity of ion channels (Na^+^, K^+^, Ca^2+^, and Mg^2+^), and ratio of energy substrate transporters in membranes [61,63]. Moreover, the consumption of deuterium-depleted drinking water by mice prevents behavioral, transcriptional, and proliferative changes typical of a depression-like state. The consumption of deuterium-depleted water also leads to electroencephalography changes observed during sleep, which resemble the effects of norepinephrine and serotonin reuptake inhibitors [64].

Several works [65,66,67,68,69,70] indicate the multidirectional effect of isotope exchange reactions on the functional activity of biological systems, their native properties, and structural organization. Nevertheless, most bioeffects associated with changes in the isotope composition of biogenic elements in the body are still not fully understood [71], which primarily concern the effect of low (compared to the natural level) concentrations of heavy nonradioactive isotopes on living systems. The latter is often associated with the traditional explanation of kinetic isotope effects, which is based on the idea of an increase in their intensity proportional to the concentration of heavy isotopes. In this case, the isotopic effects associated with a deliberate decrease in the concentration of heavy nonradioactive isotopes in comparison with their natural content, arising in complexly organized living systems during the formation of various isotope gradients, are often not fully considered [72]. According to the data available on this topic, among the studies describing changes in the ratio of the isotopes of nutrient elements, most studies pertain to the effect of various concentrations of deuterium on the body, which is explained by more pronounced isotope differences in the masses of the ^1^H and ^2^H nuclei compared to the analogous atomic weight ratio parameters of stable isotopes of oxygen, carbon, and nitrogen. However, despite the increasingly large number of studies on the effect of low δ^2^H values on living systems, researchers mostly consider changes in the ^2^H/^1^H isotope ratio in blood plasma and do not pay enough attention to comparative studies on δ^2^H values in body tissues and fluids during abundant consumption of deuterium-depleted water with modified isotope compositions [73].

In general, the effect of deuterium-depleted water on the isotope composition of tissues and morphofunctional indices in multicellular organisms is understudied; nevertheless, this topic is of particular interest, as the morphofunctional status is one of the most meaningful indicators of the development of individual organisms and their health conditions. Further, the consumption of deuterium-depleted water affects the adaptive capacity of organisms in different periods of ontogeny. It is the choice of the object of research for properly assessing the multifaceted effects of isotope exchange reactions on biological systems that is important in such scientific studies; therefore, in scientific literature, one can find studies of various unicellular and multicellular organisms [74,75,76,77,78], but often in a discrete form, without taking into account the genetic heterogeneity of individuals. However, recently the latter seems to be a relevant factor, because the different sensitivity was shown of animals of different stocks (Wistar and random bred albino male rats) in different periods of ontogeny to the effects of the drinking diet with deuterium-depleted content (δ^2^H = −705‰ [79]), including their morphofunctional indices and biochemical processes (e.g., hydrogen peroxide production by isolated mitochondria of rat liver). Besides, the isotope effect associated with a decrease in deuterium in the environment is not only expressed in animal cells but also in plant cells (e.g., deuterium-depleted water (δ^2^H = −839‰)) prevents the appearance of hyperhydricity in red beet [80].

However, the increase of ^2^H_2_O in living systems also leads to various effects [81]. Therefore, in the mice, which received deuterium (up to 10% ^2^H_2_O) as part of a drinking diet for 5 weeks and who were inoculated with _4_T1 mammary carcinoma after third week consumption ^2^H_2_O, the median survival rate was 4 days longer than that in the control group. However, of interest is the fact that deuterium-depleted water (δ^2^H were equaled from −807‰ to −358‰) was capable of causing cell cycle arrest in the G1/S transition and reducing the number of the cells in the S phase, while the superoxide dismutase and catalase activities were increased. The populations of cells in the G1 phase in MCF-7 cells were also significantly increased [82]. Therefore, such a generally similar effect on different living systems of two opposing processes (for example, the abovementioned processes associated with both increasing and decreasing ^2^H in media) requires the creation of a new hypothesis that can explain why both reduction and increase of concentrations of the same isotope can lead to significantly changes in the activity of biological substances, cells, and organisms in approximately the same amount under certain conditions (such as on the border of melting ice [83]).

## 5. ^2^H/^1^H Exchange Effects and Other Heavy Nonradioactive Isotopic Fractionations in Living and Natural Systems via Neutron Effect Realization (Hypothesis)

Apart from the abovementioned thermodynamic and kinetic effects, in the presence of light isotopes of biogenic elements, there is possibly a mechanism that is connected with the quantum tunneling effect of some heavy isotopes, as well as of covalently bound atoms of light and heavy isotopes. Comparative analysis of some experimental results from different scientific groups revealed the following regularity (Table 1): “Isotopic shock” is observed mostly when the formation of chemical bonds with an odd number of neutrons (with a non-compensated neutron, firstly by mass and charge of proton, and secondly by mass of another neutron) is highly probable, or when the system contains a chemical element (usually some metal), which also has one or several (odd number) non-compensated neutrons. The probability of such a regularity, hereinafter referred to as the neutron Basov–Dzhimak hypothesis (BADz phenomenon), was analyzed; possible mechanisms for its implementation may be connected with the ability of a non-compensated neutron to modify nuclear spins in atoms and consequently influence the reactivity of a chemical bond formed by isotopes containing non-compensated neutrons.

Based on the above discussions, we could specify the following conditions for the occurrence of the neutron BADz phenomenon in biological systems:

(1) The neutron BADz phenomenon in biological systems is observed when there are chemical bonds between the atoms showing complete predominance of neutrons over protons, marked by odd positive numbers (1, 3, 5, 7, etc.); Nn − Np = 2k + 1, where k ϵ Z (k is the integer, Z is the set of non-negative integers), N denotes number, n denotes neutrons, and p denotes protons;

(2) The neutron BADz phenomenon is observed in biological systems with chemical bonds between atoms having a fractional resultant nuclear spin, given as follows: 

(a) 2k ≠ A-spin + A’-spin ≠ 2k + 1, where k ϵ Z, A-spin denotes atomic spin of one of the isotopes involved in the formation of the chemical bond, A’-spin denotes atomic spin of another of the isotopes involved in the formation of the same chemical bond; 

(b) 2k ≠ A-spin ≠ 2k + 1, where k ϵ Z, A-spin denotes atomic spin of individual isotope without any chemical (covalent) bond with other chemical elements;

(3) The neutron BADz phenomenon is observed in biological systems where there are chemical bonds between the atoms with nuclear spins of opposite signs, as follows: R-spin: ^− +^, where “+” and “–” show the parity of two nuclei involved in the formation of the same chemical bond;

(4) Isotopic resonance intensity slightly decreases under each isolated condition (postulates), which are presented below:

Nn − Np = 2k + 1 >> 2k ≠ A-spin + A’-spin ≠ 2k + 1 >> R-spin: ^− +^

where k ε Z, N denotes number, n denotes neutrons, p denotes protons, “+” and “−” show the parity of two nuclei involved in the formation of the same chemical bond.

(5) A nonadditive amplification of isotopic resonance can be expected if any two conditions (postulates) among the first three conditions listed above are paired (points 1 and 2a, 1 and 3, or 2a and 3);

(6) The most favorable conditions for maximum isotopic resonance in biological systems are the first three of the abovementioned conditions (postulates 1–3), which were confirmed via experimental evaluations [84];

(7) In metal atoms (Me), in the absence of covalent or coordinate bonds, isotopic resonance is proportional to the properties of the metal atom (i.e., the difference between its neutrons and protons, nuclear spin, and parity of the nucleus).

It is possible to explain the mechanism of the BADz phenomenon by a violation of the mass balance in systems with equilibrium charge.

Charge equilibrium in an atom is achieved through the interaction of protons and electrons:

q [p^+^ + e^−^] = 0 (equilibrium of charge).

Mass equilibrium in an atom is achieved by the interactions of protons with neutrons and those of paired neutrons:

a.m. [p^+^ + n^0^] ≈ 1:1 (equilibrium of atomic mass),

a.m. [n_i_^0^ + n_i+1_^0^] = 1:1 (equilibrium of atomic mass).

In triads containing a proton, electron, and neutron, one can observe charge and mass equilibriums. The stronger interactions between charged particles (protons and electrons) compared to mass effects explain the absence of mass imbalance in the nuclei of atoms with a smaller number of neutrons than protons. At the same time, the presence of a neutron that is not compensated in mass can cause a mass-dependent imbalance in systems with equilibrium charge, which is characteristic of some heavy isotopes or bonds formed by them:

[p^+^ + e^−^ + n_i_^0^] · n_i+1_^0^ = 0, which is equilibrium of charge.

However, this pattern is observed ([p^+^ + e^−^ + n_i_^0^] · n_i+1_^0^) ≠ equilibrium of mass (such as 1_proton_:1_neutron_); that is, non-equilibrium of mass (in model with non-compensated neutron or neutrons: 1_proton_:1_neutron_ + odd neutron or neutrons).

A neutron not compensated in mass (n_i+1_^0^) does not affect the whole nucleus at once, but probably affects each triad (proton–electron–neutron) stochastically in time; this causes the mass effect (which is proportional to at least one-half of the equilibrium proton–neutron interactions), leading indirectly to a change in the forces of the interactions between charged particles (proton–electron interactions). The latter is confirmed by the fact that it is the hydrogen bonds that are extremely susceptible to the electron density distribution throughout the molecule [94,95]; therefore, local attenuation and amplification of the proton–electron interactions can cause tunnel effects [96].

In addition, in a system with three neutrons, the abovementioned effect can also arise because of the incapability of neutron pairs to compensate for mass fluctuations of a non-compensated neutron in an atom; however, in isotopes containing at least five more neutrons than the number of protons, partial mass equilibrium can occur because of the manner of distribution of these neutrons, which can decrease the intensity of the BADz phenomenon.

Another possible mechanism capable of significantly increasing the rate of enzyme reactions is the ability of a neutron not compensated in mass to initiate quantum tunneling by involving one of the above-described atomic triads in this process; there is a subsequent release of energy sufficient to form a new chemical bond, which can dramatically accelerate the process of substrate formation necessary for the growth of cellular structures and the overall development of organisms. This effect offers an explanation for the “isotopic shock” phenomenon observed in living systems with heavy nonradioactive isotopes of certain macro- and microelements, as well as the exponential enhancement of these manifestations when combining different isotope fractions in biological objects.

Everything stated above is highly possible, as there are certain examples of collective proton tunneling in some systems (a Zundel-like complex, [HO⋅⋅⋅D⋅⋅⋅OH]) [97]; this is because for some electron–nuclear magnetic interactions in particular electron spins, selectivity is observed with regard to the nuclear spin.

It should be noted that in living systems constituting parts of organic molecules, the BADz phenomenon will occur not in pure (isolated) isotopes, but within groups of atoms bound by covalent and noncovalent interactions. Therefore, the BADz phenomenon should be calculated at least for each atomic pair with overlapping electronic clouds. In this regard, a linear increase in the weight of isotopes that form a chemical bond will cause no linear increase in the BADz phenomenon (for example, in a ^13^C–^1^H bond, the neutron number is equal to 0 and there is no probability of isotope resonance [98]; in a ^13^C–^2^H bond, the neutron number is equal to 1, and the occurrence of isotopic resonance is highly probable (i.e., BADz phenomenon takes place); in an ^18^O–^1^H bond, where the neutron number equals 1, isotopic resonance is expected, whereas in an ^18^O–^2^H bond, the neutron number is equal to 2, and isotopic resonance is not expected). This can explain the ambiguous results of studies carried out by many researchers on the enrichment of biological systems with heavy isotopes and their mixtures [99]. The enrichment of ^13^C and ^2^H cancer cells, when the formation of isotope resonance pairs is expected (the neutron number for ^13^C–^2^H equals 1), can be considered as proving the BADz phenomenon in practice. Similar fractionations of ^12^C/^13^C and ^1^H/^2^H with the accumulation of heavy atoms are accompanied by an additional advantage in terms of the energy and metabolism in oncocytes compared to cells with natural isotope compositions (the neutron number for ^12^C–^1^H equals –1, i.e., BADz phenomenon is not observed). 

Further, an increase in the bond energy and oscillation frequency of a nucleus accompanied by a decrease in the internuclear distance can occur at different rates and with different intensities in bonds [100] formed by isotopes containing neutron pairs (^12^C–^2^H, ^18^O–^2^H) and those containing a non-compensated neutron (^13^C–^2^H). After some time, the difference between the initial energy and that observed before tunneling increases. The restriction of freedom in covalently bound resonance pairs of atoms (having a non-compensated neutron) causes an increase in the internal atomic energy, which ensures bond breakage without the necessity of achieving activation energy. This can explain the occurrence of the BADz phenomenon as one of the tunnel effect mechanisms for enzyme catalysis.

Based on the above discussions, it can be concluded that isotope fractionation in biological systems is only a prerequisite for the occurrence of isotope resonance; this resonance is observed only when the BADz phenomenon is realized, being associated with the process of incorporating heavy isotopes into biological molecules, and above all, their interaction with other light and heavy isotopes. Therefore, if the medium is excessively enriched with heavy isotopes only, the BADz phenomenon, and consequently isotopic resonance, cannot be observed in the following pairs: ^18^O–^2^H, ^34^S–^2^H, ^13^C–^15^N, ^12^C–^18^О, ^13^C–^17^О, ^14^N–^18^O, and ^15^N–^17^O.

When the concentration of deuterium in the environment is low, the body requires a much higher accumulation of ^13^C and ^15^N [87,101,102] than ^18^O and ^34^S to realize the required isotopic effect and for survival (i.e., δ^13^C _(organism)_ >> δ^13^C _(environment)_ and δ^15^N _(organism)_ >> δ^15^N _(environment)_, whereas δ^18^O _(organism)_ ≤ δ^18^O _(environment)_ [103,104,105,106], and similarly for δ^34^S _(organism)_ ≤ δ^34^S _(environment)_). In addition, a more pronounced isotopic resonance effect of ^17^O and ^18^O compared to ^13^C and ^15^N [84] can be caused by a more active participation of oxygen atoms in functionally active groups (R–^17/18^O–^1^H) in comparison with nitrogen and carbon atoms. Therefore, modification of the natural medium by enriching it with ^18^O will lead to an increase in catalytic activity or transcription rates, accompanied by an increase in the number of metabolically active molecules in cells (enzymes) or a qualitative increase in their resistance to adverse intracellular effects (cellular stress); however, enrichment of the natural medium through the introduction of ^13^C and ^15^N into its composition is more likely to be accompanied by a smaller isotopic resonance due to slowing down of the nonspecific decomposition of molecular structures upon isotopic weighting of some chemical groups (heterocycles, isoprenoids, condensed polycyclic and polyunsaturated structures, and noncovalent interactions in macromolecular complexes). In this regard, the possibility of isotopic shocks is high in metabolically active cells and tissues, for example, during their fission and growth; however, these shocks occur only in those cases where white noise, in terms of the amplitude of energy oscillations, accounts for approximately 50% of the energy potential of cells, thus initiating the occurrence of stochastic resonance. Therefore, the same isotope can resonate at different phases of the cell cycle, but it would only take place at different isotope concentrations and with different intensities of resonance, or vice versa; the same isotope pair would inhibit cellular effects upon exposure that was too weak (e.g., less than 40%, that significantly less above-mentioned 50% required for resonance phenomenon, i.e., <<50%) or too strong (more than 60% or >>50%). This also explains the ability of some combinations of isotopes in a specific cell cycle to cause super resonance, a phenomenon described by Zubarev [84].

Despite the fact that monitoring the growth of bacteria is limited by the range of temperatures of their vital activity, this effect can be explained by the following experimental data that explore the temperature effect on the growth rate of *E. coli* on the culture media with ^13^C enrichment. It was grown in a temperature ranging from 15 °C to 41 °C. It was noted that the maximum growth rates of *E. coli* were always higher in the ^13^C enrichment media than in the isotopically normal media [84]. 

This generally confirms the possibility of realization of the neutron effect (BADz phenomenon) due to electron density delocalization in the nuclei of heavy nonradioactive isotopes, even with zero (and also other integers) values of nuclear spin and the absence of magnetic moments.

Moreover, the known facts of selective isotope accumulation in biological objects in comparison with the medium, caused by the interaction of different nonradioactive isotopes with each other leading to the formation of resonance pairs, can serve as indirect proof of the BADz phenomenon. For example, at high concentrations of deuterium in the environment, in order to realize the necessary isotopic effect and to survive, the body requires much higher accumulation of ^18^O and ^34^S than ^13^C and ^15^N (i.e., δ^18^O _(organism)_ > δ^18^O _(environment)_ and δ^34^S _(organism)_ > δ^34^S _(environment)_, whereas δ^13^C _(organism)_ ≈ δ^13^C _(environment)_; similarly, δ^15^N _(organism)_ ≈ δ^15^N _(environment)_ [102]). Correspondingly, more pronounced negative correlations between δ^13^C and δ^2^H, δ^15^N, and δ^2^H are expected, while between δ^18^O and δ^2^H, as well as δ^34^S and δ^2^H, weak positive correlations or absence thereof will be observed [107]. Also, this is confirmed by some data about human infant ability to assimilate more ^13^C content and more ^15^N in fingernails than food sources contain [108], and excrete less ^2^H and ^18^O in urine [109].

The difference in the resonance effects shown by Zubarev [84], which is characterized by a 3-fold effect that is greater for ^13^C compared to ^15^N, can be explained (under the condition of equally pronounced neutron BADz phenomenon) by a much higher ability of the organism to absorb ^13^C from the enriched ^13^C diet (43.2–60.8% of the 80% enriched ^13^C diet [110], and also in [111]), compared to its ability to absorb ^15^N from the enriched ^15^N-diet (by 250% from 95% enriched ^15^N-diet [99,112]). At the same time, the co-enrichment of the medium with ^13^C and ^15^N, on the contrary, reduces the growth rate of the living organisms (for example, saprotrophic zygomycete fungus *Absidia cylindrospora* [113] or *Poecilia reticulata* guppies [86]), which is explained by the absence of a non-compensated neutron effect in these biological systems. This is because each isotope has 1 non-compensated neutron, which together form a complete pair of neutrons that do not allow the realization of the BADz phenomenon. However, in [114], it was shown that the parthenogenic rotifer *Brachionus plicatilis* receiving ^15^N resonance diet had higher fitness with a longer lifespan and similar or greater lifetime fecundity as compared to all other treatments, including the control group.

Another example is the fractionation of isotopes of various metals when they interact with individual isotopes of nonmetals. In this case, the BADz phenomenon will be realized, both in the presence of stable Me–O bonds, including coordination bonds, and in the absence of stable covalent Me–O bonds, for example in structures such as Me^+^...O^−^. The reactions based on the realization of the isotope BADz phenomenon will occur due to predominant participation of a metal ion [32,115] and its own non-compensated neutron (or, more rarely, an odd number of neutrons). For example, owing to the fact that the neutron BADz phenomenon in CuCO_3_ compounds is realized via ^63/65^Cu–^17/18^O bonds, the accumulation in this salt of both ^17^O [116] and ^18^O isotopes is expected, and therefore the ratio [δ^17^O_(organism)_/δ^17^O_(environment)_] can be slightly bigger or even equal (i.e., ≥) to the ratio [δ^18^O_(organism)_/δ^18^O_(environment)_], unlike ^40^CaCO_3_, in which ^17^O will be predominantly accumulated (i.e., ratio [δ^17^O_(organism)_/δ^17^O_(environment)_] can be significantly lager (>>>) than ratio [δ^18^O_(organism)_/δ^18^O_(environment)_]). In ^43^CaCO_3_, the differences in the isotopic ratios of ^17^O and ^18^O will be substantially less pronounced due to the fractionation of both atoms; for example, the ratio [δ^17^O_(organism)_/δ^17^O_(environment)_] can be moderately larger or even equal (> or ≥) to ratio [δ^18^O_(organism)_/δ^18^O_(environment)_].

Thus, the created hypothesis explains the BADz phenomenon through the occurrence of isotopic resonance, both with decreasing and increasing ^2^H, and through other non-radioactive isotopes in living and natural systems due to the appearance of resonant pairs between chemical bound atoms, whose interaction result in an uncompensated neutron. 

## 6. Possibility of Using the Isotopic Therapy in ^2^H-Modified Drinking and Diet for Some Diseases

Using knowledge of isotope resonance through the BADz phenomenon will also allow more efficient poly-isotope correction of metabolic disorders in various diseases by creating conditions for targeted fractionations of isotopes in certain pathological cells and certain focuses of the disease. The latter is relevant because in current medical practice, increasing numbers of different metabolic disorders and cell pathologies are corrected by mostly monoisotopic medicines, especially ^2^H-isotope-modified drinking and diet therapies in healing the following diseases and certain conditions:

− Alzheimer’s disease was treated by deuterium-reinforced polyunsaturated fatty acids (D-PUFAs), which improved cognition in mice fed a Western-type diet [117], and reduced the brain tissue concentrations of oxidation products and amyloid β-peptide in APP/PS1 mutant transgenic mice [118];
− Parkinson’s disease was partially cured by the administration of D-PUFAs, which protected against nigrostriatal damage (in the substantia nigra) from oxidative injury in mice with a 1-methyl-4-phenyl-1,2,3,6-tetrahydropyridine model [119];
− Mitochondrial disorders and oxidative stress were corrected by both deuterium-depleted water [16,55,56,65,69,120,121] and D-PUFAs, such as 11,11-D(2)-linoleic and 11,11,14,14-D(4)-α-linolenic acids and others, which preserved mitochondrial bioenergetics function under toxic effects of *t*-butylhydroperoxide, ethacrynic acid, or ferrous iron [122], prevented coenzyme Q (ubiquinone or CoQ)-less coq mutants and some toxic effects of lipid autoxidation products [123,124,125], and also decelerated the aging process by deuterated trilinolenin (D-TG (54:9)), resulting in extended lifespan of worms under normal and oxidative stress conditions [126];
− Cerebral hypoxia was corrected by deuterium-depleted water, the long-term consumption of which resulted in activation of neuron protective systems, with higher antioxidant activity in brain tissues [68,75];
− Friedreich’s ataxia was treated by deuterated linoleic and α-linolenic acids, which resulted in rescue of oxidative-stress-challenged cells by decreasing lipid peroxidation [127], and also deuterated ethyl linoleate (RT001), which recovered mitochondrial function [128];
− Huntington’s disease was managed by D-PUFA diet in Q140 knock-in mice, which were able to demonstrate improved performance in novel object recognition tests after 5 months of consumption of deuterium-enriched diet [129];
− Cancer adjuvant therapy showed efficacy through the administration of ^1^H_2_O [22,70,130,131], and previously-drug-processed gliomas was treated by combining deuterium-depleted drinking and a lower deuterated diet, which led to intracellular deuterium disequilibrium and formation of the ^2^H/^1^H gradient [62,132];
− Inflammatory diseases were fine-tuned to potentially various inflammation stages using deuterated isotopologues of eicosanoids (for example, arachidonic acids with ^2^H at the bis-allylic C_7_, C_10_, and C_13_ positions [133]);
− Pathological signaling pathways in diabetes were corrected by deuterium-depletion in drinking and diet [17,134,135];
− Cardiovascular diseases were treated by improving cholesterol handling and reducing atherosclerosis development using D-PUFAs, which decreased lipid peroxidation, body weight gain, and plasma and hepatic cholesterol contents [136], as well as by using D-PUFAs to reduce the tocopherol-mediated peroxidation of lipids in human low-density lipoproteins [137].

Moreover, some effects of deuterium-reinforced amino acids (DRAAs) in the pathological pathways were recently shown (especially under the conditions of deficiency of the corresponding enzyme), such as procollagen-lysine 5-dioxygenases [138], protein-lysine 6-oxidase [139], and DNA methyltransferase 3A [140]. Also, synthesized (in vitro) hexapeptide containing asparaginyl residue with three carbon-bound atoms replaced with deuterium atoms (by reducing the speed of racemization) stabilized polypeptides, limiting the accumulation of some of the peptides and proteins in aging products [141]. 

The powerful effects of the organism of the selective ^2^H-enriched pharmaceuticals on biochemical potency are not known, which enable substantial benefits in the treatment of some diseases [142], but according to another study, livestock products were more effective in the therapy of cyclophosphamide-induced immunodeficiency after extraction of immunoactive proteins by deuterium-depleted media [52]. This points to a similar effect of heavy-isotope fluctuations, relating frequently to both their increase and decrease [37,143,144], with approximately the same number of fluctuations influencing living systems due to isotopic discrimination “up and down” in different stages of the lifespan [109]. Considering the phenomenon of isotope resonance in living systems can expand the healing possibilities of adjuvant and other types of therapies via reasonable poly-isotopic correction, this could be more effective than monoisotopic treatment.

## 7. Conclusions

In conclusion, it should be noted that the intensity of the isotope resonance can vary depending on the concentrations of the isotopes [144]: at low concentrations of potentially resonant isotopes (e.g., due to interaction deuterium with atoms such as ^13^C, ^15^N, and ^17^O), their thermodynamic and kinetic effects are predominantly realized (which are characterized by relatively insignificant differences in the fractionation rates), whereas at high concentrations of the same isotopes, the probability of the resonant pair formation with the subsequent appearance of the valence isotope BADz phenomenon, allowing for implementation of tunneling, causes anomalous (or paradoxical) isotope effects in the same biological reactions.

Although the monoisotopic-modified drinking and diet showed some efficacy in treating many pathologies (especially by changing the ^2^H/^1^H ratio in cells using deuterium depleted water, D-PUFAs, or DRAAs), such as Alzheimer’s disease, Friedreich’s ataxia, Huntington’s disease, brain cancer, cardiovascular and inflammatory diseases, and mitochondrial disorders, including Parkinson’s disease, diabetes, and cerebral hypoxia, polyisotopic resonance therapy in living systems could become a more effective type of therapy than the monoisotopic treatment.

In addition, it is important to emphasize that in living systems, the phenomenon known as “isotopic shock” can be realized by forming an isotope gradient that stimulates the work nonspecific protection system, leading to the accumulation of biologically active protective factors in the body (e.g., intracellular messengers and reactive oxygen species). These factors, together with the thermodynamic and kinetic effects described above, as well as neutron tunneling, are the driving forces causing a pronounced variety of isotopic compositions observed in biological objects, depending not only on the isotope composition of the medium but also on the functional activity of the organism itself, as well as peculiarities in the interactions of different isotopes among themselves when fractionated at different morphofunctional levels in the biological object itself. 

The timeline of the reviewed papers is from 1960 to 2019. 

## Figures and Tables

**Table 1:** Isotopic resonance regularity in biological and natural systems occurring because of the neutron Basov–Dzhimak hypothesis in individual chemical bonds and some individual isotopes.

I	II	III	IV	V	VI	VII	VIII	IX	X
^12^C-^1^H		^12^C-^2^H							
6n − 7p	−1	7n − 7p	0						
R-spin	0^+^ & ½^+^ is ½^+ +^	R-spin	0^+^ & 1^+^ is 1^+ +^						
		^13^C-^1^H [84]		^13^C-^2^H [41]					
		7n − 7p	0	8n − 7p	1				
		R-spin	½^−^ & ½^+^ is 1^− +^	R-spin	½^−^ & 1^+^ is 1½^− +^				
^14^N–^1^H		^14^N-^2^H							
7n − 8p	−1	8n − 8p	0						
R-spin	1^+^ & ½^+^ is 1½^+ +^	R-spin	1^+^ & 1^+^ is 2^+ +^						
		^15^N-^1^H [84]		^15^N-^2^H					
		8n − 8p	0	9n − 8p	1				
		R-spin	½^−^ & ½^+^ is 1^− +^	R-spin	½^−^ & 1^+^ is 1½^− +^				
^16^O-^1^H		^17^O-^1^H		^18^O-^1^H [84]					
8n − 9p	−1	9n − 9p	0	10n − 9p	1				
R-spin	0^+^ & ½^+^ is ½^+ +^	R-spin	5/2^+^ & ½^+^ is 3^+ +^	R-spin	0^+^ & ½^+^ is ½^+ +^				
		^16^O-^2^H		^17^O-^2^H		^18^O-^2^H			
		9n − 9p	0	10n − 9p	1	11n − 9p	2		
		R-spin	0^+^ & 1^+^ is 1^+ +^	R-spin	5/2^+^ & 1^+^ is 3½^+ +^	R-spin	0^+^ & 1^+^ is 1^+ +^		
^32^S-^1^H		^32^S-^2^H		^34^S-^1^H		^34^S-^2^H			
16n − 17p	−1	17n − 17p	0	18n − 17p	1	19n − 17p	2		
R-spin	0^+^ & ½^+^ is ½^+ +^	R-spin	0^+^ & 1^+^ is 1^+ +^	R-spin	0^+^ & ½^+^ is ½^+ +^	R-spin	0^+^ & 1^+^ is 1^+ +^		
		^12^C-^12^C		^12^C-^13^C					
		12n − 12p	0	13n − 12p	1				
		R-spin	0^+^ & 0^+^ is 0^+ +^	R-spin	0^+^ & ½^−^ is ½^+ −^				
		^12^C-^14^N		^12^C-^15^N					
		13n − 13p	0	14n − 13p	1				
		R-spin	0^+^ & 1^+^ is 1^+ +^	R-spin	0^+^ & ½^−^ is ½ ^+ −^				
				^13^C-^14^N		^13^C-^15^N [84,85,86]			
				14n − 13p	1	15n − 13p	2		
				R-spin	½^−^ & 1^+^ is 1½ ^+ −^	R-spin	½^−^ & ½^−^ is 1 ^− −^		
		^12^C-^16^О		^12^C-^17^О		^12^C-^18^О			
		14n − 14p	0	15n − 14p	1	16n − 14p	2		
		R-spin	0^+^ & 0^+^ is 0^+ +^	R-spin	0^+^ & 5/2^+^ is 5/2^+ +^	R-spin	0^+^ & 0^+^ is 0^+ +^		
				^13^C-^16^О		^13^C-^17^О		^13^C-^18^О [84]	
				15n − 14p	1	16n − 14p	2	17n − 14p	3
				R-spin	½^−^ & 0^+^ is ½^− +^	R-spin	½^−^ & 5/2^+^ is 3^− +^	R-spin	½^−^ & 0^+^ is ½^− +^
		^14^N-^16^O		^14^N-^17^O		^14^N-^18^O			
		15n − 15p	0	16n − 15p	1	17n − 15p	2		
		R-spin	1^+^ & 0^+^ is 1^+ +^	R-spin	1^+^ & 5/2^+^ is 3½^+ +^	R-spin	1^+^ & 0^+^ is 1^+ +^		
				^15^N-^16^O [87]		^15^N-^17^O		^15^N-^18^O [84]	
				16n − 15p	1	17n − 15p	2	18n − 15p	3
				R-spin	½^−^ & 0^+^ is ½ ^− +^	R-spin	½^−^ & 5/2^+^ is 3 ^− +^	R-spin	½^−^ & 0^+^ is ½^− +^
		^40^Ca [27]						^43^Ca [27]	
		20n − 20p	0					23n − 20p	3
		R-spin	0^+^					R-spin	7/2^−^
		^24^Mg [29]		^25^Mg [29,88]		^26^Mg [29]			
		12n − 12p	0	13n − 12p	1	14n − 12p	2		
		R-spin	0^+^	R-spin	5/2^+^	R-spin	0^+^		
								^63^Cu-^18^O	
								44n − 37p	7
								R-spin	3/2^−^ & 0^+^ is 3/2^− +^
								^63^Cu	
								34n − 29p	5
								R-spin	3/2^−^
								^65^Cu	
								36n − 29p	7
								R-spin	3/2^−^
		^28^Si [89]		^29^Si [89]		^30^Si [89]			
		14n − 14p	0	15n − 14p	1	16n − 14p	2		
		R-spin	0^+^	R-spin	1/2^+^	R-spin	0^+^		
						^64^Zn [90,91]		^67^Zn [91,92]	
						34n − 30p	4	37n − 30p	7
						R-spin	0^+^	R-spin	5/2^−^
						^66^Zn [90,91]			
						36n − 30p	6		
						R-spin	0^+^		
						^238^U [93]		^235^U [93]	
						146n − 92p	54	143n − 92p	51
						R-spin	0^+^	R-spin	7/2^−^

Note: n—neutron; p—proton; D—deuterium; R-spin—resultant spin of individual isotopes or pair of isotopes with chemical bonds (as a sum of individual spins); k—integer. In the table, all references [27,29,41,84,85,86,87,88,89,90,91,92,93] confirm the new hypothesis and arising resonance in living and natural systems only under certain isotopes. Note: I, III, and VII—pairs of isotopes with chemical bonds and individual isotopes that must have no isotopic resonance; II—there is no isotopic resonance for: Nn − Np = −1; IV—there is no isotopic resonance for: Nn − Np = 0; V and IX—pairs of isotopes with chemical bonds and individual isotopes that have some isotopic resonance; VI—there is some isotopic resonance for: Nn − Np = 1; VIII—there is no isotopic resonance for: Nn − Np = 2, and Nn − Np = 2k; X—there is some isotopic resonance for: Nn − Np = 2k + 1; N—number.
